# Supplementary material for: Understanding community member and health care professional perspectives on gender-affirming care—A qualitative study
Source: PLoS One. 2021 Aug 16;16(8):e0255568. doi: 10.1371/journal.pone.0255568 (PMC8366980; doi:10.1371/journal.pone.0255568)
Supplement: S1 Appendix — (DOCX) [file pone.0255568.s001.docx]

**PATH Project (Plan and Act for Transgender Health)**

**Interview Script- Providers**

1. **INTRODUCTION**

**Introduce self and other team members in the room**

Hello, my name is [name of interviewer]. Thank you for agreeing to participate in this interview.

- The goal of this study is to learn about the lived experiences, physical and mental health, and unmet health care and social service needs of transgender children, adolescents, and adults and families in Western Massachusetts and other parts of New England and New York.
- Other aims of the study include; estimating the size of the transgender population in these areas; mapping health care and social service organizations who provide services to transgender people; and gathering perspectives from providers and communities of transgender people about how health care and social services can be improved.

**Explain the purpose of the interview**

- Your interview will help us to better understand the challenges and benefits of future programs to improve the delivery of health and social services for people who are transgender in Western Massachusetts and the surrounding areas. We will be interviewing different people from your organization and other organizations in the area to gain multiple perspectives.

**Describe the audio recording and how we will assure confidentiality and answer any questions:**

- This interview will be audio recorded
- Please be assured that the audio files will be kept confidential. Leadership in your organization and any other co-workers will not have access to any of your responses and they will not be able to connect your responses to you personally. We will remove any information or examples from our records that might identify your identity. Only members of our research team will hear the interviews and the audio recording will be destroyed as soon as a transcript is verified and analyzed by the research staff.
- If, at any time, you feel that the questions are too sensitive, I would be happy to turn off the recorder during that portion of questioning. You may also skip any questions you wish during the interview.

**Review consent criteria:** Your participation in this interview study is completely voluntary. This means you do not have to participate if you don’t want to. If you agree to participate, you have the right to only answer the questions you choose to answer. Participation in the study takes approximately 90 minutes. You also have the right to stop participation at any point during the study if you so choose.

The format of the study today is a one-to-one interview. I/we will be conducting the interview today.

The potential risks of this research are minimal and confidentiality of private health information that you share with us will be maintained to the highest level. Your name will not be connected in any way with your responses. This study is approved with a Waiver of Documentation of Consent. This means that you will not be asked to formally sign a consent form that links your name to the study. Instead, we are asking for your verbal affirmation that you consent to participate.

You should be aware that in some situations the law might require us to release your health information without your permission. For example, in the event that they hear about situations of abuse, researchers and health care workers are required to report abuse or neglect of children to the Department of Children, Youth and Families, and to report abuse or neglect of people age 60 and older to the Department of Elderly Affairs.

Although there are no direct benefits to you for participating in the study, your responses will help us to learn about gaps and barriers to health and social services for transgender people and also help to inform the design of how these services are delivered in the future. You will receive a $50 gift card for your time and participation in the interview. Do you have any questions for me? [Answer any questions]

**Would you like to proceed?**

*If yes, continue.*

Thank you for agreeing to participate in this interview, we really appreciate your time.

Are you ready to begin? I’m going to start recording now.

Do I have your consent to audio record this interview?

*If yes, continue.*

1. **BACKGROUND INFORMATION**

I am going to ask you a series of open-ended questions. I want to hear your thoughts so please do not hesitate to share whatever you believe might be related to any of the topics.

**First, I would like to ask you a few questions to help me understand your role in this organization.**

**Please describe your role in the organization.**

*PROBES*

- How do you interact with patients or clients?
- What are your main responsibilities?
- How is your time allocated?

1. **EXISTING SERVICES**

**What are the current policies and procedures for the care of transgender people in your organization?**

**What kind of gender affirming care and specialty services do you already offer patients/clients who receive services in your organization?**

*PROBES*

- Please describe the different services/programs specific to the transgender population
- Please describe policies or procedures that are followed by specialty departments to provide gender affirming care.
- To what extent are clients/patients referred to services outside of your organization? (Describe services/resources)

**What are your procedures / processes for culturally tailoring and linguistically tailoring services for specific populations you serve? Please provide an example.**

**How knowledgeable would you say your staff are in serving trans / non-binary people?**

**What gaps in knowledge and skills do you see for your staff related to trans/ non-binary care?**

**What kind of training is already offered to staff and administration on gender identity and inclusive practices for a gender diverse community?**

*PROBES*

- How is the training received? (frequency, reviews of material)
- Is the training mandatory for all employees?
- What topics are included in the training?

**Patient navigation and case management are used for many different patient populations. Are these services available for transgender/non-binary clients specifically?**

**What holistic wellness services does your organization currently provide? Are these services available for transgender/non-binary clients specifically?**

**Do you currently offer a trauma-informed service environment? If so, has this trauma-informed approach been tailored for transgender and non-binary clients?**

**Please describe your current practices for the collection of data related to gender identity.**

*PROBES*

- Do you collect gender identity data in registration forms, and if so, how?
- Do you record gender identity data in client records, and if so, how?
- Do you collect and record gender identity information about employees, and if so, how?
- Do you collect gender identity information in the employment application process, and if so, how?

**What is your organization’s policy to ensure that hiring practices are inclusive?**

**How does your organization currently engage with a local community which is gender diverse?**

*PROBES*

- How is gender diversity affirmed in the waiting room and at check in?
- How does your outreach strategy facilitate interaction with gender diverse community members?

**IV. Perceived Barriers to Care/Implementation of services**

**What do you think are the barriers to gender affirming care in your organization?**

**What are specific financial and insurance barriers related to services for transgender individuals?**

**What ideas or interventions has your organization used to try to address those barriers?**

*PROBES*

• How have they worked?

•What has worked best? Why?

•What didn’t work? Why?

**How could your organization be more inclusive and affirming as it pertains to the gender identity of both employees and community members accessing services?**

**What type of interventions, including training or policy changes, would be needed in your organization to best meet the needs of the transgender population?**

**How would your organization decide whether or not to adopt this type of intervention?**

**How would you personally be involved in this process?**

*PROBES*

•Who would participate in the decision-making process?

•Would you say the implementation of a new program is typically more externally driven, meaning it follows a directive from management, or is usually internally motivated, meaning staff feels the need for a new program? How (if at all) does that influence the implementation of the program?

•Do you feel that interventions to improve the quality of care for the transgender community will be fully supported by the senior leadership of your organization?

1. **IMPLEMENTATION**

**Now, I would like to hear about the process you would go through to get these services implemented.**

**Will you please describe how services or policy changes would be implemented in your organization?**

*PROBES*:

- Who would be responsible for the different roles involved?
- How could this program get buy-in from staff?
- How could this program get buy-in from management?
- What are the important outcomes to be measured and how would these outcomes be measured?

**What type of technical assistance would facilitate the implementation, success, and sustainability of this program or policy change?**

*PROBES*:

- With what frequency would you or your organization need to access technical assistance in the form of webinars, in-person trainings, or calls?

1. **COSTS**

**What kind of funding support would be needed to implement specialty services for transgender and non-binary clients in your organization?**

*PROBES***:**

- What would be the cost of having dedicated staff time for appropriate personnel to serve transgender and non-binary clients?
- What are any additional financial barriers to implementing specialty services for transgender and non-binary clients?

**That’s all the questions I have for you. Is there anything I’ve missed or anything you’d like to add?**
