# Supplementary material for: Understanding community member and health care professional perspectives on gender-affirming care—A qualitative study
Source: PLoS One. 2021 Aug 16;16(8):e0255568. doi: 10.1371/journal.pone.0255568 (PMC8366980; doi:10.1371/journal.pone.0255568)
Supplement: S3 Appendix — (DOCX) [file pone.0255568.s003.docx]

**PATH Project (Plan and Act for Transgender Health)**

**Focus Group Guide- Healthcare Administrators, Providers and Front-Line Staff**

**I. INTRODUCTIONS**

Hello, my name is [name of moderator]. Thank you for agreeing to participate in this focus group.

This focus group discussion will help us to better understand the challenges and benefits of future programs to improve the delivery of health and social services for people who are transgender in Western Massachusetts and the surrounding areas. Your thoughts and point of view are important and that is why you have been asked to participate. I know you are busy and I appreciate your time.

**Audio Recording:**

We are recording this focus group in order to ensure that the content is captured fully. Although it will be taped, please be assured that the tapes will be kept confidential.

- We will remove any information or examples from our records that might identify your identity. Only members of our research team will hear the interviews and the audio recording will be destroyed as soon as a transcript is verified and analyzed by the research staff.

**Review consent criteria**

Your participation in this focus group is completely voluntary. This means you do not have to participate if you don’t want to. If you agree to participate, you have the right to only answer the questions you choose to answer. Participation in the study takes approximately 90 minutes. You also have the right to stop participation at any point during the study if you so choose.

The format of the study today is a focus group. I will be moderating the discussion today.

**Risks**

Some of the questions in the focus group are related to the experience of discrimination some people may have had while accessing health or social services. There is some risk that discussing or hearing about these events could trigger an emotional response during your participation in this study. You are free to refuse to answer any question and may end your participation in the study at any time. The researchers have a list of local or accessible online resources that we can provide to you if you become upset during focus group participation, and any information you disclose to the researcher will remain confidential.

You should be aware that in some situations the law might require us to release your health information without your permission. For example, in the event that they hear about situations of abuse, researchers and health care workers are required to report abuse or neglect of children to the Department of Children, Youth and Families, and to report abuse or neglect of people age 60 and older to the Department of Elderly Affairs.

Although there are no direct benefits to you for participating in the study, your responses will help us to learn about gaps and barriers to health and social services for transgender people and also help to inform the design of how these services are delivered in the future. Do you have any questions for me? [Answer any questions]

**Would you like to proceed?**

*If yes, continue.*

**Ground rules**

- Everyone’s experience is valid and there are no right or wrong answers.
- You do not have to speak in any particular order
- Please, only one person at a time
- Although we may not agree with everything that is said, we can respect that person’s experience.
- What is shared in this group stays in the group
- We will be recording so we don’t miss anything
- Please keep everything you hear today confidential.
- We will summarize the themes of the discussion without identifying individuals by name.

Does anyone have any questions? (answer any questions).

Ok, let’s begin.

**Introductory Question**

Please take a few moments to think about your organization’s current policies and procedures for the care of transgender people. How is the care you provide gender-affirming?

**Guiding Questions**

*EXISTING SERVICES*

What kind of training is already offered to staff and administration on gender identity and inclusive practices for a gender diverse community?

- How is the training received? (frequency, reviews of material)
- Is the training mandatory for all employees?
- What topics are included in the training?

Patient navigation and case management are used for many different patient populations. Are these services available for transgender/non-binary clients specifically?

What holistic wellness services does your organization currently provide? Are these services available for transgender/non-binary clients specifically?

Do you currently offer a trauma-informed service environment? If so, has this trauma-informed approach been tailored for transgender and non-binary clients?

Please describe your current practices for the collection of data related to gender identity.

- Do you collect gender identity data in registration forms, and if so, how?
- Do you record gender identity data in client records, and if so, how?
- Do you collect and record gender identity information about employees, and if so, how?
- Do you collect gender identity information in the employment application process, and if so, how?

What is your organization’s policy to ensure that hiring practices are inclusive?

How does your organization currently engage with a local community which is gender diverse?

- How is gender diversity affirmed in the waiting room and at check in?
- How does your outreach strategy facilitate interaction with gender diverse community members?

*BARRIERS AND IMPLEMENTATION*

What do you think are the barriers to gender affirming care in your organization?

What are specific financial and insurance barriers related to services for transgender individuals?

What ideas or interventions has your organization used to try to address those barriers?

*PROBES*

• How have they worked?

•What has worked best? Why?

•What didn’t work? Why?

How could your organization be more inclusive and affirming as it pertains to the gender identity of both employees and community members accessing services?

What type of interventions, including training or policy changes, would be needed in your organization to best meet the needs of the transgender population?

How would your organization decide whether or not to adopt this type of intervention?

**Concluding question**

- Of all the things we’ve discussed today, what would you say are the most important issues you would like to express?

**Conclusion**

- Thank you for participating. Your thoughts and ideas are a valuable asset to the study. I would like to remind you that any comments featuring in this report will be anonymous. Please remember to keep the contents of this discussion confidential. If you have any further questions or concerns please feel free to contact me.
