# Supplementary material for: Understanding community member and health care professional perspectives on gender-affirming care—A qualitative study
Source: PLoS One. 2021 Aug 16;16(8):e0255568. doi: 10.1371/journal.pone.0255568 (PMC8366980; doi:10.1371/journal.pone.0255568)
Supplement: S4 Appendix — (DOCX) [file pone.0255568.s004.docx]

**PATH Project (Plan and Act for Transgender Health)**

**Focus Group Guide- Parents**

**I. INTRODUCTIONS**

Hello, my name is [name of moderator]. Thank you for agreeing to participate in this focus group.

This focus group discussion will help us to better understand the challenges and benefits of future programs to improve the delivery of health and social services for people who are transgender in Western Massachusetts and the surrounding areas. Your thoughts and point of view are important and that is why you have been asked to participate. I know you are busy and I appreciate your time.

**Audio Recording:**

We are recording this focus group in order to ensure that the content is captured fully. Although it will be taped, please be assured that the tapes will be kept confidential.

•We will remove any information or examples from our records that might identify your identity. Only members of our research team will hear the interviews and the audio recording will be destroyed as soon as a transcript is verified and analyzed by the research staff.

**Review consent criteria:**

Your participation in this focus group is completely voluntary. This means you do not have to participate if you don’t want to. If you agree to participate, you have the right to only answer the questions you choose to answer. Participation in the study takes approximately 90 minutes. You also have the right to stop participation at any point during the study if you so choose.

The format of the study today is a focus group. I will be moderating the discussion today.

**Risks**

Some of the questions in the focus group are related to the experience of discrimination some people may have had while accessing health or social services. There is some risk that discussing or hearing about these events could trigger an emotional response during your participation in this study. You are free to refuse to answer any question and may end your participation in the study at any time. The researchers have a list of local or accessible online resources that we can provide to you if you become upset during focus group participation, and any information you disclose to the researcher will remain confidential.

You should be aware that in some situations the law might require us to release your health information without your permission. For example, in the event that they hear about situations of abuse, researchers and health care workers are required to report abuse or neglect of children to the Department of Children, Youth and Families, and to report abuse or neglect of people age 60 and older to the Department of Elderly Affairs.

Although there are no direct benefits to you for participating in the study, your responses will help us to learn about gaps and barriers to health and social services for transgender people and also help to inform the design of how these services are delivered in the future. Do you have any questions for me? [Answer any questions]

Would you like to proceed?

*If yes, continue.*

**Ground rules**

- Everyone’s experience is valid and there are no right or wrong answers.
- You do not have to speak in any particular order
- Please, only one person at a time
- Although we may not agree with everything that is said, we can respect that person’s experience.
- What is shared in this group stays in the group
- We will be recording so we don’t miss anything
- Please keep everything you hear today confidential.
- We will summarize the themes of the discussion without identifying individuals by name.

Does anyone have any questions? (*answer any questions*).

Ok, let’s begin.

Introductory question

Please take a moment to think about the various health and social services you may have accessed for your child recently. This could be a pediatrician, specialty provider, therapist, mental health provider, urgent care facility or emergency room. How have these experiences been gender-affirming? How have they not?

Guiding questions

**EXISTING SERVICES**

- What services/organizations offer gender-affirming care for your child and are already available in your community?
- How often do you access these services?
- What health services are available through your child’s school? How do they support your child in a way that is gender-affirming?
- What services exist in the community to help you in accessing or advocating for gender-affirming care for your child?
- When or how often do you access services specific to helping you navigate health systems for gender-affirming care for your child?
- What services for holistic care and wellness (e.g., massage, reiki, alternative & complementary medicine) offer gender-affirming care?

**BARRIERS**

- What barriers have you encountered in attempting to access excellent gender-affirming pediatric care for your child?
- What do you think are the main barriers or difficulties that families with transgender children have when accessing health or social services?
- In what ways has your child’s health care been affected (if at all) by issues of transportation? (This refers to access to affordable, reliable ways of traveling to and from medical appointments.)
- In what ways has your child’s health been affected (if at all) by limited access to food or healthy foods?
- How has living in a rural area impacted your child’s health or ability to access health services?
- Was there ever a time that you were afraid to access health care for your child because you were worried they would not receive gender-affirming care?

**IMPLEMENTATION**

- What kind of resources would be helpful for you when you are navigating health systems?
- What do you think about the idea of having different specialties that are gender-affirming and in one location?
- Do you think behavioral health or mental health services should be integrated in the same location as primary care or should they be kept separate?

**Concluding question**

Of all the things we’ve discussed today, what would you say are the most important issues you would like to express?

**Conclusion**

Thank you for participating. Your thoughts and ideas are a valuable asset to the study. I would like to remind you that any comments featured in this report will be anonymous. Please remember to keep the contents of this discussion confidential. If you have any further questions or concerns please feel free to contact me.
